# How socio-emotional learning in physical education curriculum affects student well-being: a moderated mediation analysis

**DOI:** 10.3389/fpsyg.2026.1835014

**Published:** 2026-06-19

**Authors:** Jie Zhang

**Affiliations:** Department of Physical Education, Zhumadian Preschool Education College, Zhumadian, Henan, China

**Keywords:** emotional regulation, moderated mediation, physical education, school climate, socio-emotional learning, student well-being

## Abstract

**Introduction:**

Socio-emotional learning (SEL) has been increasingly recognized as a key contributor to students’ well-being, particularly when integrated into school curricula such as physical education (PE). Drawing on the CASEL framework, Self-Determination Theory, and Ecological Systems Theory, this study examines how SEL embedded in PE influences student well-being, focusing on the mediating roles of emotional regulation and peer relationship quality, as well as the moderating effect of school climate.

**Methods:**

Data were collected from 452 high school students (aged 14–18) across three provinces in China using validated self-report measures. Confirmatory factor analysis (CFA) was conducted to establish construct validity, followed by multilevel path modeling to test a moderated mediation model.

**Results:**

Results indicated that SEL was significantly associated with student well-being through emotional regulation in a manner consistent with the hypothesized indirect relationship, whereas peer relationship quality did not serve as a significant mediator. Moreover, the indirect effect of SEL on well-being via emotional regulation was significantly moderated by school climate, with stronger effects observed in more supportive environments. Bootstrapping analyses confirmed a significant total indirect effect through emotional regulation, while the pathway via peer relationships remained non-significant.

**Discussion:**

These findings are consistent with the view that emotional regulation represents an important psychological pathway associated with the relationship between SEL in PE and student well-being, and that a positive school climate enhances this effect. The study underscores the importance of integrating SEL within supportive school contexts to optimize its impact on adolescent well-being.

## 1 Introduction

Socio-emotional learning (SEL) has gained global prominence as an essential dimension of holistic education, equipping students with the skills needed to manage emotions, build healthy relationships, and make responsible decisions (Mondi et al., 2021). While the impact of SEL has been extensively documented in relation to academic success and behavioral adjustment, its implications for mental well-being, particularly in subject-specific contexts such as physical education (PE), remain underexplored (Simion, 2023). Mental well-being is conceptualized in this study as a state of positive psychological functioning encompassing emotional balance, resilience, and adaptive coping, consistent with the operational focus of school-based well-being research (Sorrenti et al., 2025). PE offers a uniquely emotionally and socially dynamic environment, where students regularly engage in teamwork, conflict, success, and failure—conditions that naturally stimulate socio-emotional processes. Yet, SEL research has largely concentrated on core academic subjects, leaving a critical gap in understanding how these competencies translate within PE settings where physical interaction and social engagement are highly salient (Chang and Tsai, 2022). Although prior SEL studies have examined emotional and relational pathways associated with student well-being (Morcillo-Martínez et al., 2026; Sindiani et al., 2025), comparatively limited research has investigated whether these mechanisms operate differently within PE contexts characterized by heightened emotional exposure, physical performance, and peer interaction. Accordingly, the present study seeks to extend existing SEL literature by examining whether the relative importance of intrapersonal and interpersonal mechanisms differs within activity-based educational environments such as PE.

Among the key psychological mechanisms theorized to link SEL with student well-being, emotional regulation stands out as a consistently validated predictor. Importantly, emotional regulation skills developed through repeated and emotionally salient PE activities are not expected to remain context-bound, but rather to generalize to broader psychological functioning, including students’ overall sense of mental well-being and future-oriented optimism. Physical education provides frequent exposure to emotionally charged situations (Simion, 2023; Chang and Tsai, 2022)—such as competition, cooperation, success, and failure—that serve as practice contexts for regulatory skills that can be applied beyond the PE setting. Adolescents face growing emotional demands in school, and the ability to regulate emotions such as frustration, anxiety, and excitement is vital for maintaining mental well-being (Váradi, 2022). SEL programs explicitly aim to foster such self-management skills, which are considered foundational for resilience and adaptive functioning (Demirci et al., 2022). At the same time, peer relationship quality also deserves theoretical and empirical attention, as peer acceptance, support, and belonging are deeply intertwined with adolescent well-being (Allbright and Hough, 2020). SEL interventions often aim to improve relationship skills, yet research has rarely examined how peer dynamics in PE environments specifically mediate the SEL–well-being link. There is a need to concurrently test these two distinct but interrelated pathways—intrapersonal (emotional regulation) and interpersonal (peer relationships)—to understand their unique contributions to student well-being. In particular, examining both pathways simultaneously allows the present study to clarify whether emotional or relational competencies constitute the more influential mechanism linking SEL with well-being in PE settings, thereby extending prior SEL mediation models that have generally treated these competencies as uniformly beneficial.

Moreover, while individual-level mechanisms are essential, school climate plays a critical contextual role in shaping the success of SEL programs. A positive school climate, characterized by emotional safety, supportive teacher-student relationships, and respectful peer interactions, creates fertile ground for SEL competencies to develop and thrive (Hoskins and Schweig, 2024). In the present study, socio-emotional learning, emotional regulation, and peer relationship quality are conceptualized as PE-contextualized student-level constructs, reflecting students’ experiences during physical education classes, whereas school climate is intentionally modeled as a broader, whole-school contextual factor operating at the institutional level. Previous research has shown that supportive school climates strengthen student engagement, emotional adjustment, and social–emotional functioning across general educational settings (Yang et al., 2020; Jones et al., 2020). Within PE contexts specifically, emotionally supportive and inclusive environments may be particularly important because PE activities frequently involve public performance, peer evaluation, teamwork, and competitive interaction, all of which intensify students’ emotional and social experiences (Dyson et al., 2021). These characteristics suggest that school climate may shape how effectively SEL-related emotional and relational competencies are experienced and expressed during PE participation. Yet, limited research has examined how school climate interacts with SEL at a cross-level, particularly within PE, to influence emotional and social development. Moreover, multilevel designs that incorporate school-level moderators are necessary to examine how environmental conditions may be associated with variations in the relationships between SEL and student outcomes (Yang et al., 2020). Understanding these interactions is especially crucial in collectivist cultures like China, where institutional factors often shape behavioral norms and emotional expression (Liang et al., 2022; Ryan and Deci, 2000). Thus, beyond examining direct and indirect associations, the present study contributes by investigating whether supportive school environments strengthen the effectiveness of SEL-related emotional and relational processes in PE contexts.

To guide this inquiry, the present study draws upon three complementary theoretical perspectives. The Collaborative for Academic, Social, and Emotional Learning (CASEL) framework provides the competency-based structure of SEL, delineating emotional regulation and peer relationship skills as core developmental capacities. Self-Determination Theory (SDT) explains how these competencies support mental well-being by facilitating adaptive need-related functioning, particularly perceptions of competence and relatedness in learning environments (Sorrenti et al., 2025; Niemiec and Ryan, 2009). Meanwhile, Ecological Systems Theory (Bronfenbrenner, 1979) supports the investigation of school climate as a contextual moderator, positing that students’ developmental outcomes emerge from interactions between individual capacities and institutional environments. Together, these frameworks are integrated to examine whether SEL is associated with mental well-being, but also through which mechanisms and under what school-level conditions these effects are strengthened or constrained. Integrating these theoretical lenses, the study proposes a multilevel moderated mediation model in which SEL is associated with student well-being through both emotional regulation and peer relationships, and where school climate moderates these effects.

**Figure 1:**
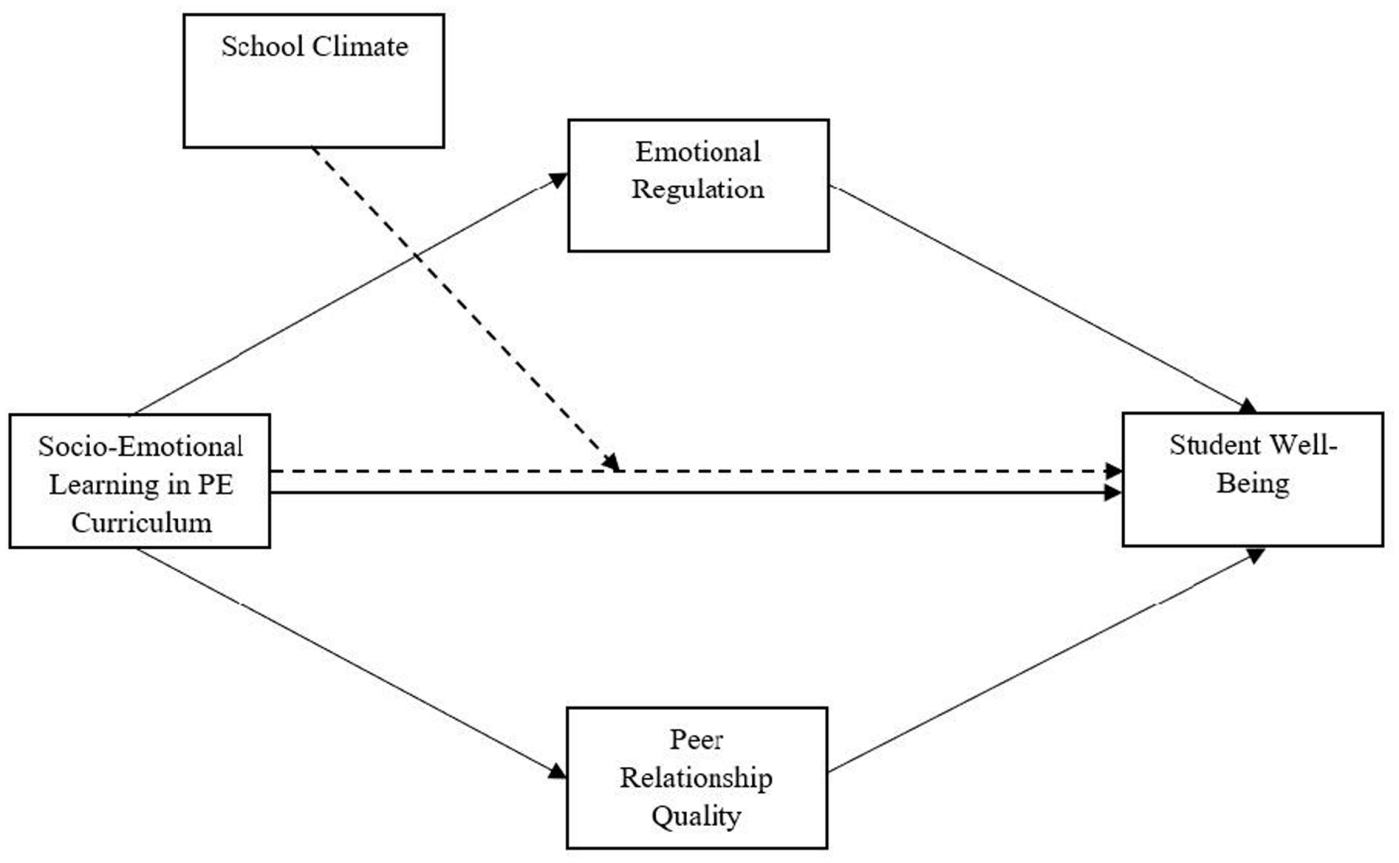
Proposed multilevel moderated mediation framework showing student-level (Level 1) and school-level (Level 2) relationships. Dashed arrows represent cross-level moderation and direct associations.

This study addresses important gaps by (1) investigating SEL in a non-academic subject (PE), (2) comparing two distinct mediating pathways—emotional and social—within a single explanatory model, and (3) explicitly accounting for school-level contextual influences through multilevel analysis. More importantly, the study extends prior SEL mediation research (León et al., 2023; Wang et al., 2026) by demonstrating that the mechanisms linking SEL with student well-being may function differently within emotionally and socially dynamic PE environments. Specifically, the findings reveal that emotional regulation represents a more central pathway connecting SEL with well-being than peer relationship quality, highlighting the differential importance of intrapersonal and interpersonal processes in PE settings. In addition, by modeling school climate as a cross-level contextual condition, the study further clarifies how supportive institutional environments may strengthen the effectiveness of SEL-related emotional competencies. Accordingly, the contribution of the present study lies not only in the PE-specific context, but also in clarifying differential pathway effects and the contextual conditions under which SEL-related processes become more effective. In doing so, it expands current understandings of how, through what mechanisms, and under what conditions SEL contributes to adolescent mental well-being (Figure 1).

## 2 Literature review

SEL has become an integral focus of educational research, policy, and practice over the past two decades due to its potential to enhance students’ emotional, social, and academic outcomes (Durlak et al., 2011; Taylor et al., 2017). Rooted in the CASEL framework, SEL encompasses five interrelated competencies: self-awareness, self-management, social awareness, relationship skills, and responsible decision-making (Collaborative for Academic, Social, and Emotional Learning, 2020). These competencies have been consistently linked to positive student outcomes, including increased academic achievement, reduced behavioral problems, and enhanced mental well-being (Sorrenti et al., 2025). Among these competencies, self-management—specifically emotional regulation—has received considerable attention as a key mechanism linking SEL to mental health and well-being outcomes (Váradi, 2022). Simultaneously, relationship skills such as forming and maintaining supportive peer connections are also central to adolescent development and mental well-being (You et al., 2023). The present study aims to investigate the effects of SEL in physical education (PE) settings on student well-being, with a focus on the mediating roles of emotional regulation and peer relationship quality, and the moderating role of school climate. This literature review elaborates on the theoretical foundations and empirical support for each proposed pathway.

### 2.1 Socio-emotional learning and student well-being

The role of SEL in shaping adolescent mental well-being is now firmly grounded in educational literature. Large-scale syntheses such as Pérez and Bahamon Muneton (2023) and Winardi et al. (2022) consistently show that school-based SEL interventions not only enhance academic performance but also reduce psychological distress and promote emotional resilience. These effects are particularly pronounced during adolescence, a developmental period characterized by heightened emotional sensitivity and social dependence (Mengíbar et al., 2025). Recent cross-cultural investigations also lend support; for instance, Ayllón-Salas and Fernández-Martín (2024) found that SEL practices significantly predicted life satisfaction and self-esteem among Chinese secondary students, reinforcing SEL’s relevance across educational systems.

Theoretically, the CASEL, 2020 framework positions SEL as a foundation for lifelong social and emotional functioning. It is designed to cultivate competencies like self-awareness and responsible decision-making, which have direct implications for mental well-being. These competencies are believed to satisfy key psychological needs outlined in Self-Determination Theory (Sorrenti et al., 2025)—namely, autonomy, competence, and relatedness—each empirically linked to psychological well-being (García-Martínez et al., 2023). In parallel, ecological approaches (Bronfenbrenner, 1979) emphasize that psychological development is a product of interaction between internal competencies and environmental experiences. SEL enhances this interactional capacity, enabling students to engage more adaptively within their social and academic microsystems.

In PE contexts, these competencies take on particular importance. PE exposes students to emotionally charged scenarios—winning, losing, cooperation, and public performance—that test both intrapersonal and interpersonal skills. When equipped with SEL competencies, students navigate these situations with greater emotional control and social ease, ultimately contributing to improved mental well-being (Váradi, 2022). Given this multidimensional alignment, SEL in PE is expected to predict students’ overall psychological well-being.

*H1*: Socio-emotional learning in physical education positively predicts student well-being.

### 2.2 Mediating role of emotional regulation

A considerable body of empirical work identifies emotional regulation as a central mechanism through which SEL influences mental well-being. Emotional regulation—defined as the capacity to monitor, evaluate, and modulate emotional responses (Li et al., 2023)—has been consistently shown to buffer stress and promote adaptive functioning (Törmänen et al., 2023). A systematic review by Rodríguez et al. (2022) revealed that strategies like reappraisal and acceptance significantly predicted lower symptoms of anxiety and depression, particularly during adolescence. More recently, Serey et al. (2025) demonstrated that emotional regulation fully mediated the relationship between school-based SEL exposure and subjective well-being among Chinese students. Within the CASEL model, emotional regulation is a cornerstone of the self-management competency. SEL curricula explicitly target this domain through techniques such as emotion labeling, breathing strategies, and reflective exercises (Simion, 2023). Studies employing the RULER approach Gualda et al. (2023) show that when students gain emotional vocabulary and awareness, they experience enhanced emotion regulation, which in turn reduces behavioral issues and promotes mental well-being.

From a motivational perspective, Self-Determination Theory proposes that emotional regulation enhances students’ perceived competence—one of the three psychological nutrients essential for sustained mental well-being (Ryan and Deci, 2000). Students who feel capable of regulating frustration, anxiety, and embarrassment in demanding settings such as PE are more likely to remain engaged and emotionally stable. Within PE contexts, these regulatory capacities are repeatedly activated through performance pressure, peer evaluation, and competitive interaction, making emotional regulation a particularly salient intrapersonal pathway. Cultural studies further suggest that in collectivist societies where emotional restraint is socially valued, emotional regulation plays an especially critical role in adaptive functioning (Hayashi et al., 2022). Accordingly, emotional regulation is expected to serve as a key explanatory pathway linking SEL to student well-being.

*H2*: Emotional regulation mediates the relationship between socio-emotional learning and student well-being.

### 2.3 Mediating role of peer relationship quality

Adolescence is not only a period of heightened emotionality but also of intensified social engagement (Serey et al., 2025). The quality of peer relationships during this period exerts a powerful influence on students’ self-concept, mental health, and sense of school belonging (LaBelle, 2023). SEL programs, particularly those emphasizing relationship-building and empathy, are designed to enhance peer connectivity through direct instruction and structured social interactions (Collaborative for Academic, Social, and Emotional Learning, 2020). For example, Zheng (2022), in a multi-school study, found that SEL program participants reported more positive peer relationships and reduced social conflict.

Unlike the intrapersonal function of emotional regulation, peer relationship quality reflects students’ interpersonal integration within the school ecology. According to SDT, relatedness—the feeling of being connected to and cared for by others—is a non-negotiable component of mental well-being (Sorrenti et al., 2025). Students embedded in warm peer networks tend to show more engagement, fewer internalizing symptoms, and greater resilience in the face of academic or social stressors (You et al., 2023). Indeed, a recent meta-analysis by Moreira et al. (2021) confirmed that perceived peer support is a robust predictor of student well-being across educational levels.

In PE specifically, where cooperative gameplay and social dynamics are central, peer relationships serve both as a source of motivation and as a regulatory mechanism for behavior (Slee and Skrzypiec, 2016). A student who feels excluded or judged may disengage from PE altogether, while those with strong peer bonds often report higher enjoyment and sustained participation. These dynamics underscore the importance of peer relationships as a mediating pathway in the SEL–well-being link.

*H3*: Peer relationship quality mediates the relationship between socio-emotional learning and student well-being.

### 2.4 Moderating role of school climate

While SEL equips students with essential emotional and relational competencies, the extent to which these skills are effectively applied and internalized is shaped by the broader educational environment—most notably, the school climate. School climate is commonly conceptualized as a multidimensional construct encompassing emotional safety, the quality of interpersonal relationships, fairness in discipline, student engagement, and support from teachers and staff (Wang and Degol, 2016). In recent years, research has shifted from treating school climate as a static background condition to viewing it as an active moderator of intervention effects, particularly in the domain of SEL (Allbright and Hough, 2020). Meta-analytic findings affirm the role of school climate in enhancing student outcomes. For instance, Berkowitz et al. (2017) synthesized results from 78 studies and found that the presence of a positive school climate strengthened the impact of SEL interventions on students’ emotional and behavioral development. Similarly, Yang et al. (2020) emphasized that SEL programs are more successful when implemented in environments where students feel emotionally secure and relationally supported. When schools provide students with consistent emotional cues, trustworthy adult relationships, and institutional norms that encourage respectful interaction, SEL skills are more likely to be practiced, reinforced, and generalized (Collie et al., 2011).

Theoretically, school climate operates as a proximal environmental influence within Bronfenbrenner’s ecological systems theory (Bronfenbrenner, 1979), directly shaping how internal competencies—such as emotional regulation and peer relationship quality—are developed and expressed. In line with the proposed multilevel model, school climate is expected to condition the strength of the indirect pathways linking SEL to student well-being, rather than functioning as a direct predictor. In emotionally supportive school climates, students receive both the psychological safety to practice regulation strategies and the social reinforcement necessary to develop quality peer relationships (Jones et al., 2020). Conversely, climates characterized by low teacher support, high conflict, or relational disengagement may suppress these processes, thereby attenuating the benefits of SEL.

Empirical research further supports this conditional process. McCormick et al. (2015), in a large-scale multilevel analysis, showed that the benefits of SEL on self-regulation and stress management were significantly amplified in classrooms with warm teacher-student relationships and high behavioral expectations. Likewise, Yang et al. (2021) found that students in schools with higher school climate ratings exhibited stronger emotional and relational competencies following SEL participation compared to peers in lower-rated schools. Contextual factors are particularly salient in non-Western cultures, such as China, where collectivist values and educational hierarchies intensify the role of institutional environment. In such settings, school climate not only affects behavior but also shapes emotional norms, peer interactions, and receptivity to SEL content (Rivers et al., 2013). Research in Chinese secondary schools reveals that emotional openness, mutual respect, and classroom harmony significantly condition the effectiveness of emotional and social learning initiatives (Yang et al., 2018).

Given this empirical and contextual evidence, school climate is expected to moderate both indirect pathways: (1) from SEL to well-being via emotional regulation, and (2) from SEL to well-being via peer relationship quality. In supportive climates, students are more likely to implement self-regulatory strategies and experience reinforcing peer dynamics, strengthening both mediating effects. In contrast, under less supportive conditions, these indirect paths may weaken or dissipate.

*H4a*: The indirect effect of socio-emotional learning on student well-being via emotional regulation is moderated by school climate, such that the effect is stronger in schools with a more positive climate.

*H4b*: The indirect effect of socio-emotional learning on student well-being via peer relationship quality is moderated by school climate, such that the effect is stronger in schools with a more positive climate.

## 3 Methodology

### 3.1 Participants and sampling procedure

Participants for this study were drawn from public high schools across three provinces in mainland China—Jiangsu, Sichuan, and Guangdong—selected for their geographic diversity and varying school climates. A total of 452 students (aged approximately 14–18 years) enrolled in PE courses participated in the study. These students were nested within 92 public high schools, which constituted the Level-2 units in the multilevel analysis. To ensure a representative and diverse sample, a multi-stage cluster sampling approach was employed. First, schools were randomly selected from official regional education directories. Within each school, intact PE classes were chosen at random, and all students in those classes were invited to participate.

Eligibility criteria included: (1) enrollment in a compulsory PE course during the semester of data collection, (2) ability to understand and respond to a written survey in Mandarin, and (3) parental or guardian consent for minors. Participation was voluntary, and students could withdraw at any point without consequence. Of the distributed 480 questionnaires, 452 were returned fully completed, resulting in a response rate of 94.2%. Questionnaires containing substantial missing responses were excluded prior to analysis. Examination of the retained responses indicated minimal item-level missingness (<2% across all study variables). Little’s MCAR test was non-significant (*p* > 0.05), suggesting that the remaining missing data were consistent with a missing completely at random (MCAR) pattern. Given the very low proportion of missing values and the absence of systematic missingness, listwise deletion was considered appropriate and unlikely to introduce substantial estimation bias (Little and Rubin, 2020).

### 3.2 Procedure and ethical considerations

Prior to data collection, approval was obtained from the Institutional Review Board (IRB) of the lead researcher’s university. All participating schools provided administrative consent through formal letters of cooperation. To ensure ethical compliance, a cover letter accompanied each questionnaire, explaining the purpose of the study, assuring participants of the confidentiality and anonymity of their responses, and clearly stating that their participation was voluntary.

Informed consent was obtained from all participants. For students under 18, written parental consent was secured along with student assent. The study adhered to ethical guidelines outlined in the Declaration of Helsinki, including the right to withdraw, data confidentiality, and non-harmful procedures. No personally identifiable information was collected.

Data were collected during regular PE sessions to minimize disruption and ensure consistency. Students completed the questionnaire individually under the supervision of a trained school liaison and the research team, who provided clarification when needed. The average completion time was approximately 20–25 min. Data collection was conducted during the spring semester of the 2024 academic year, specifically between March and May 2024. All participating schools were surveyed within this same general time window to maintain consistency in contextual and academic conditions across institutions. Conducting the survey within a relatively uniform period helped reduce potential temporal variation in socio-emotional learning experiences, emotional regulation, and school climate perceptions across schools.

### 3.3 Demographic information

Among the 452 participants, 51.3% were female (*n* = 232) and 48.7% were male (*n* = 220). In terms of grade level, 57.1% were in lower secondary (grades 9–10) and 42.9% in upper secondary (grades 11–12). Regarding prior exposure to SEL programs, 54.9% reported having participated in structured SEL activities at least twice in the past academic year. This variable was included as a control to account for students’ previous familiarity with socio-emotional learning activities. Students came from a mix of urban (62.4%) and rural (37.6%) school contexts, allowing for some variability in environmental and institutional factors such as school climate. Although the participating schools followed nationally standardized PE curriculum guidelines, variations in instructional delivery, activity formats, and physical activity exposure across schools may still have existed due to regional and institutional differences. Accordingly, demographic and prior SEL exposure variables were included as controls to partially account for contextual variability across participating schools.

## 4 Measures

All constructs in this study were assessed using well-established and psychometrically validated scales adapted to the educational context of PE. All items were rated on a five-point Likert scale ranging from 1 (strongly disagree) to 5 (strongly agree), with higher scores indicating higher levels of the respective construct.

Socio-emotional learning was measured using a 5-item scale adapted from the SEL framework developed by Collaborative for Academic, Social, and Emotional Learning (2020) and operationalized in school-based studies such as those by Durlak et al. (2011), focusing on students’ perceptions of emotional awareness, self-management, and responsible decision-making in PE classes. A sample item is “I am able to manage my emotions effectively during PE activities.” The scale demonstrated strong internal consistency (Cronbach’s *α* = 0.81). Although CASEL represents a conceptual competency framework rather than a standalone psychometric instrument, the present study adapted item content from previously operationalized school-based SEL measures grounded in CASEL competencies, particularly those related to self-awareness, self-management, social awareness, relationship skills, and responsible decision-making (Collaborative for Academic, Social, and Emotional Learning, 2020; Durlak et al., 2011; Taylor et al., 2017). The adapted items were contextually modified to reflect students’ experiences within PE settings while preserving the conceptual meaning of the original competency domains. Prior to full-scale data collection, the items were reviewed by two educational psychology scholars and two experienced PE educators to ensure content relevance and contextual appropriateness. In addition to internal consistency, construct validity was evaluated through confirmatory factor analysis, where SEL items demonstrated satisfactory standardized factor loadings exceeding recommended thresholds and adequate discriminant validity relative to the other study constructs.

Emotional regulation was assessed using the Emotion Regulation Questionnaire for Children and Adolescents (ERQ-CA) (Gullone and Taffe, 2012), which consists of 10 items measuring cognitive reappraisal and expressive suppression. A sample item is “I control my feelings by changing the way I think about the situation.” The ERQ-CA demonstrated strong internal consistency in the present study (Cronbach’s *α* = 0.86).

Peer relationship quality was measured using 8 items adapted from the Peer Relations Scale (Bukowski et al., 1994), focusing on perceived trust, support, and acceptance from peers during physical education activities. A sample item is “My classmates support me during PE classes.” This scale showed strong reliability (Cronbach’s *α* = 0.84).

Student mental well-being was assessed using a brief version of the Warwick–Edinburgh Mental Well-being Scale (WEMWBS) (Tennant et al., 2007), consisting of 7 items capturing positive emotional functioning and psychological resilience. A sample item is “I have been feeling optimistic about the future.” Internal consistency for this scale was strong (Cronbach’s *α* = 0.91). Although the well-being items capture general psychological functioning (e.g., optimism and positive affect), the use of this scale is theoretically justified because emotional regulation—fostered through PE-based SEL—is conceptualized as a transferable capacity that supports broader mental well-being rather than context-specific mood states.

School climate, serving as a Level-2 moderator, was measured at the group level using 12 items from the School Climate Inventory developed by Wang and Degol (2016), encompassing teacher support, safety, and student engagement dimensions. Individual responses were aggregated to the school level to reflect shared climate perceptions. The aggregated scale demonstrated strong reliability (Cronbach’s *α* = 0.78). To justify aggregation of school climate to the school level, within-group agreement and reliability statistics were examined following recommendations for multilevel research (LeBreton et al., 2023). The results indicated acceptable agreement among respondents within schools (mean rwg = 0.84), supporting the assumption of shared climate perceptions. In addition, the ICC (1) value for school climate was 0.14, indicating meaningful between-school variance, while the ICC (2) value was 0.79, demonstrating acceptable reliability of the school-level means. These statistics collectively supported the aggregation of school climate as a Level-2 construct suitable for multilevel and cross-level moderation analyses.

It is important to note that all student-level constructs (socio-emotional learning, emotional regulation, peer relationship quality, and mental well-being) were measured using items explicitly framed within the physical education context, ensuring that responses reflected students’ experiences during PE classes. In contrast, school climate was deliberately operationalized as a general, school-level construct capturing shared perceptions of the broader academic and social environment, rather than PE-specific climate. This distinction aligns with Ecological Systems Theory (Bronfenbrenner, 1979), which conceptualizes school climate as a higher-order contextual system that shapes how domain-specific learning experiences, such as PE-based SEL, translate into broader developmental and well-being outcomes.

### 4.1 Analytical approach

This study employed a multilevel moderated mediation analysis to examine the underlying mechanisms through which socio-emotional learning (SEL) in the physical education (PE) curriculum affects student mental well-being. Given the nested data structure—students nested within schools—a multilevel modeling approach was adopted to accurately account for group-level variance.

Prior to hypothesis testing, intraclass correlation coefficients (ICCs) were calculated to assess the proportion of variance attributable to the school level. The ICC for student mental well-being was 0.12, indicating sufficient between-school variability to justify multilevel modeling. These ICC estimates were computed within the Mplus 8.3 environment as part of the multilevel modeling framework. Specifically, we investigated two mediating pathways—emotional regulation and peer relationship quality—while also testing the moderating role of school climate at Level 2.

To further examine the robustness of the proposed hypotheses, a series of nested model comparisons was conducted during the multilevel analysis process. Competing models were compared by sequentially examining direct-effect, mediation, and moderated mediation structures to evaluate whether the inclusion of cross-level interaction terms involving school climate improved overall model fit and explanatory power. School climate was explicitly modeled as a Level-2 contextual construct to distinguish institutional influences from student-level psychological processes.

Preliminary analyses included confirmatory factor analysis (CFA) to establish the discriminant validity of the constructs. The five-factor measurement model—comprising socio-emotional learning, emotional regulation, peer relationship quality, student mental well-being, and school climate—demonstrated superior fit compared to alternative models, supporting the hypothesized measurement structure.

Subsequent analyses involved estimating direct and indirect effects using multilevel path models via hierarchical linear modeling. All analyses were conducted using Mplus 8.3, following established procedures for multilevel moderated mediation models. Descriptive statistics were initially examined to screen the data and were subsequently verified within Mplus to ensure consistency with the multilevel analyses. Prior to hypothesis testing, missing data patterns were examined to ensure compliance with APA JARS reporting standards. Because the remaining item-level missingness was minimal and consistent with MCAR assumptions, listwise deletion was applied during model estimation. To assess mediation effects, the product-of-coefficients approach was applied, and bootstrapping with 5,000 resamples was used to estimate indirect effects and corresponding 95% confidence intervals.

## 5 Results

Table 1 presents the results of confirmatory factor analyses (CFA) comparing the baseline five-factor measurement model with several alternative, more parsimonious models. The five-factor model, which treats socio-emotional learning (SEL), emotional regulation (ER), peer relationship quality (PRQ), student well-being (SWB), and school climate (SC) as distinct constructs, demonstrated the best model fit: χ^2^(84) = 287.41, RMSEA = 0.066, CFI = 0.967, TLI = 0.951. These fit indices fall within acceptable thresholds, as RMSEA values less than 0.08 and CFI/TLI values greater than 0.90 are generally considered indicative of good model fit (Hu and Bentler, 1999).

**Table 1:** Measurement model comparison.

Model description	χ^2^	df	Δχ^2^	RMSEA	CFI	TLI
Five-factor model (baseline)	287.41	84	—	0.066	0.967	0.951
Four-factor model (merged ER & PRQ)	333.89	87	46.48**	0.075	0.942	0.923
Three-factor model (merged SEL, ER & PRQ)	359.12	89	71.71**	0.079	0.930	0.912
Two-factor model (all constructs combined)	381.66	90	94.25**	0.082	0.908	0.887

RMSEA, root mean square error of approximation; CFI, comparative fit index; TLI, Tucker–Lewis index; SEL, Socio-Emotional Learning; ER, Emotional Regulation; PRQ, Peer Relationship Quality; SWB, Student Well-being; SC, School Climate. ∗∗*p* < 0.01, two-tailed.

In contrast, alternative models that merged one or more constructs showed a decline in fit quality. The four-factor model, which combined ER and PRQ into a single factor, resulted in a significant chi-square difference (Δχ^2^ = 46.48, *p* < 0.01), along with higher RMSEA (0.075) and lower CFI (0.942) and TLI (0.923). Similar or poorer fit was observed for the three-factor and two-factor models. These results indicate that the five-factor model provided the most adequate representation of the measurement structure.

In addition, sequential multilevel model comparisons supported the inclusion of cross-level interaction effects involving school climate. Models incorporating Level-2 moderation demonstrated improved interpretive value relative to simpler direct-effect and mediation-only models, supporting the appropriateness of the proposed multilevel moderated mediation framework. These comparisons further reinforced the contextual relevance of school climate in shaping the associations between SEL-related processes and student well-being.

Table 2 presents the descriptive statistics and bivariate correlations among the key study variables. The mean scores for socio-emotional learning (M = 3.79, SD = 0.66), emotional regulation (M = 3.74, SD = 0.62), and student well-being (M = 3.72, SD = 0.59) suggest moderate to high perceived levels across the sample (*N* = 452). Correlations among variables generally align with theoretical expectations. Specifically, socio-emotional learning was significantly and positively associated with emotional regulation (*r* = 0.49, *p* < 0.01), peer relationship quality (*r* = 0.46, *p* < 0.01), and student well-being (*r* = 0.38, *p* < 0.01).

**Table 2:** Descriptive statistics and correlations analysis.

Variable	M	SD	1	2	3	4	5	6	7	8
1. Gender^a^	0.52	0.50	—							
2. Grade Level^b^	0.59	0.49	−0.04	—						
3. Past SEL Exposure^c^	0.56	0.50	−0.03	0.11*	—					
4. Socio-Emotional Learning (SEL)	3.79	0.66	−0.02	0.14**	0.17**	—				
5. Emotional Regulation (ER)	3.74	0.62	0.01	0.13*	0.16**	0.49**	—			
6. Peer Relationship Quality (PRQ)	3.66	0.65	0.03	0.09	0.14*	0.46**	0.42**	—		
7. Student Well-being (SWB)	3.72	0.59	0.05	0.07	0.12*	0.38**	0.41**	0.33**	—	
8. School Climate (SC)	2.91	0.44	−0.02	0.06	0.07	0.21**	0.20**	0.13*	0.10	—

*N* = 452. All continuous variables were grand-mean centered prior to analysis.

a Gender coded as 0 = male, 1 = female.

b Grade Level coded as 1 = higher grades (11–12), 0 = lower grades (9–10).

c Past SEL Exposure = participated in SEL-focused PE activities at least twice in past year. *p < 0.05. **p < 0.01.

Emotional regulation was also positively associated with student well-being (*r* = 0.41, *p* < 0.01), supporting its potential mediating role. While peer relationship quality was significantly correlated with well-being (*r* = 0.33, *p* < 0.01), this association was comparatively weaker. School climate was positively related to SEL (*r* = 0.21, *p* < 0.01) and emotional regulation (*r* = 0.20, *p* < 0.01), indicating that a supportive environment may enhance SEL outcomes. Control variables such as gender, grade level, and past SEL exposure showed small but statistically significant correlations with several predictors. These results provide preliminary support for the hypothesized relationships and justify the inclusion of all variables in the multilevel moderated mediation model.

The results presented in Table 3 provide robust support for the hypothesized multilevel moderated mediation model. In line with prior research (Durlak et al., 2011; Taylor et al., 2017), socio-emotional learning demonstrated a significant positive association with both student well-being and the two proposed mediators: emotional regulation and peer relationship quality. However, a deeper look at Model 4 reveals that only emotional regulation significantly mediated the relationship between SEL and student well-being (*β* = 0.19, *p* < 0.001), whereas the indirect path via peer relationship quality was not statistically significant (*β* = 0.12, ns). The moderation analysis further strengthens this interpretation. A significant cross-level interaction between emotional regulation and school climate (*β* = 0.37, *p* < 0.001) suggests that the indirect effect of SEL on well-being via emotional regulation is stronger in schools with a more supportive and inclusive climate.

**Table 3:** Multilevel moderated mediation analysis.

Predictor	M1 (SWB) β (SE)	M2 (ER) β (SE)	M3 (PRQ) β (SE)	M4 (SWB) β (SE)
Level 1 Predictors				
Intercept	3.70*** (0.08)	3.73*** (0.07)	3.65*** (0.09)	3.68*** (0.08)
Gender	0.01 (0.03)	0.04 (0.03)	−0.03 (0.04)	0.02 (0.03)
Grade Level	0.05* (0.02)	0.02 (0.03)	0.01 (0.03)	0.04* (0.02)
Past SEL Exposure	0.00 (0.03)	0.03 (0.02)	0.02 (0.03)	0.02 (0.03)
Socio-Emotional Learning	0.24** (0.05)	0.50*** (0.04)	0.47*** (0.05)	0.06 (0.04)
Level 2 Predictors				
School Climate	0.03 (0.04)	−0.02 (0.03)	−0.04 (0.04)	−0.01 (0.03)
SEL Differentiation	0.20 (0.10)	0.11 (0.09)	0.19 (0.10)	0.15 (0.08)
Cross-Level Interactions				
SEL × School Climate	0.04 (0.05)	0.13 (0.06)	−0.06 (0.05)	−0.15 (0.07)
Mediators				
Emotional Regulation	—	—	—	0.19*** (0.05)
Peer Relationship Quality	—	—	—	0.12 (0.06)
ER × School Climate	—	—	—	0.37*** (0.08)
PRQ × School Climate	—	—	—	0.03 (0.05)
Model Deviance	395.76	484.01	569.27	369.82

Standard errors are reported in parentheses. *N* = 452 individuals, 92 school units. SEL, Socio-Emotional Learning; ER, Emotional Regulation; PRQ, Peer Relationship Quality; SWB, Student Well-being; SC, School Climate. *p < 0.05, **p < 0.01, ***p < 0.001.

Table 4 presents the results of the mediation analysis using the bootstrap method, which estimates the significance of indirect effects by resampling. The results show that emotional regulation significantly mediated the relationship between socio-emotional learning and student well-being, with a strong path from SEL to emotional regulation (P_MX_ = 0.50, *p* < 0.01), and from emotional regulation to well-being (P_YM_ = 0.36, *p* < 0.01). The resulting indirect effect (0.18) was statistically significant (*p* < 0.01), confirming the mediating role of emotional regulation. When combined with the small but non-significant direct effect (P_YX_ = 0.06), the total effect of SEL on student well-being was 0.24, demonstrating that a substantial portion of SEL’s influence on well-being operates through emotional competencies.

**Table 4:** Estimating mediation analysis using bootstrap procedure.

Mediator	PMX	PYM	Direct effect (PYX)	Indirect effect	95% Bootstrap CI	Total effect
Emotional regulation	0.50**	0.36**	0.06	0.18**	[0.10, 0.27]	0.24**
Peer relationship quality	0.47**	0.19	0.06	0.09	[−0.02, 0.18]	0.15

*N* = 452. P_MX_, path from socio-emotional learning in PE curriculum to mediator; P_YM_, path from mediator to student well-being. *p < 0.05. **p < 0.01.

**Figure 2:**
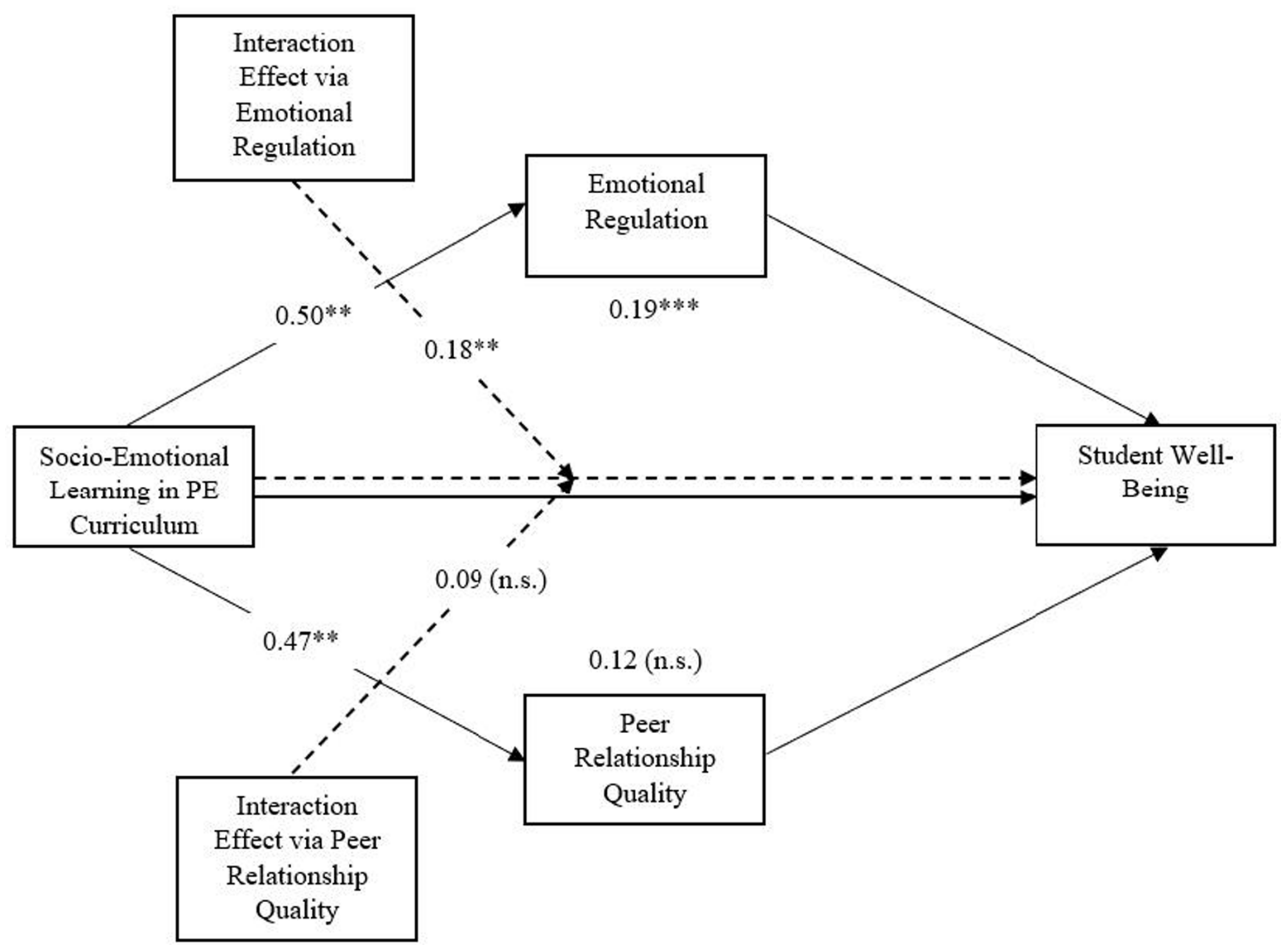
Multilevel structural model with direct, indirect, and cross-level interaction paths.

In contrast, peer relationship quality (PRQ) did not significantly mediate the SEL–well-being link. Although SEL was significantly related to PRQ (P_MX_ = 0.47, *p* < 0.01), the path from PRQ to well-being (P_YM_ = 0.19) did not reach significance, and the indirect effect (0.09) was also non-significant. These results reinforce findings from the moderated mediation model (Table 3; Figure 2), where emotional regulation emerged as the primary pathway linking SEL to positive well-being outcomes.

## 6 Discussion

The present study examined how socio-emotional learning (SEL) embedded within the physical education curriculum relates to student mental well-being by simultaneously testing two mediating mechanisms—emotional regulation and peer relationship quality—and the moderating role of school climate. Consistent with prior evidence highlighting the psychosocial benefits of SEL (Durlak et al., 2011; Taylor et al., 2017), the findings indicate that SEL is indirectly associated with student mental well-being, but that this relationship operates primarily through emotional regulation rather than peer relationship quality. This pattern underscores the importance of distinguishing between intrapersonal and interpersonal pathways when evaluating the effectiveness of SEL interventions in activity-based educational contexts such as physical education. By moving beyond a single-path explanation, the present findings contribute to a more differentiated understanding of how SEL functions within emotionally and socially dynamic learning environments.

### 6.1 Emotional regulation as a central pathway linking SEL and well-being

Emotional regulation emerged as the central mediating mechanism linking SEL to student mental well-being. This finding is consistent with previous research suggesting that emotional self-regulation may function as a proximal psychological resource that enables students to cope effectively with stress, performance pressure, and social evaluation in school settings (Rivers et al., 2013). In physical education contexts, students are frequently exposed to emotionally salient situations involving competition, public performance, and peer comparison. SEL competencies associated with emotional regulation may therefore be more closely linked immediate and consistent influence on mental well-being by supporting emotional stability and adaptive coping during these experiences. The strong indirect effect observed through emotional regulation suggests that SEL initiatives in PE may be particularly effective when they prioritize self-management skills that help students regulate emotional responses in demanding situations. These findings support the view that PE-based SEL contributes to students’ mental well-being indirectly by strengthening emotional regulation capacities that generalize beyond the PE context, thereby influencing broader indicators of psychological functioning such as optimism and emotional balance. This interpretation extends existing SEL research by clarifying that PE serves not merely as a physical activity context, but also as an emotionally rich training ground for transferable self-regulatory skills.

### 6.2 Differential role of peer relationship quality

In contrast, peer relationship quality did not significantly mediate the relationship between SEL and student mental well-being, despite being positively associated with SEL. This finding diverges from some prior research that emphasizes the protective role of peer support for adolescent well-being (Moreira et al., 2021). One plausible explanation is that peer relationship quality may function as a more distal or context-dependent resource, whose effects on well-being are less direct than those of emotional regulation. In collectivist and academically competitive contexts, such as many Chinese secondary schools, peer relationships may be shaped by hierarchical norms, social comparison, and performance-oriented expectations, potentially limiting their capacity to serve as stable emotional buffers (Low and Van Ryzin, 2024). Additionally, during early and middle adolescence, students may still be developing the social maturity required to form deeply supportive peer bonds, rendering intrapersonal regulatory capacities more influential for well-being during this developmental stage. Thus, the absence of a significant peer-mediated pathway should not be interpreted as a lack of social relevance, but rather as an indication that emotional regulation may play a more foundational role in supporting well-being within PE contexts.

### 6.3 School climate as a contextual condition shaping SEL effectiveness

The moderating role of school climate further refines the interpretation of these findings. The results indicate that a supportive school climate significantly strengthened the indirect effect of SEL on mental well-being through emotional regulation, but not through peer relationship quality. This pattern is consistent with ecological perspectives emphasizing that individual competencies are activated and reinforced within supportive institutional environments (Foster et al., 2022; Trach et al., 2018). When schools provide emotionally safe, supportive, and respectful climates, students may feel more secure in practicing emotional regulation strategies acquired through SEL, thereby amplifying their benefits for mental well-being. Prior research similarly suggests that positive school climates enhance the effectiveness of SEL programs by reinforcing emotional norms, teacher support, and shared values that promote self-regulatory development (Yang et al., 2020; Wang and Degol, 2016; Jones et al., 2020).

Importantly, PE teachers likely play a central role in shaping how SEL-related experiences are translated into students’ emotional and psychological outcomes within PE settings. Unlike many traditional classroom environments, PE classes involve continuous social interaction, public performance, cooperation, competition, and immediate emotional feedback, placing PE teachers in a particularly influential position regarding students’ emotional experiences. Teachers who encourage supportive communication, inclusive participation, constructive feedback, and cooperative engagement may help students feel psychologically safe while navigating emotionally demanding activities. In such environments, students may become more willing to practice emotional regulation strategies during competitive or socially evaluative situations, which may help explain the stronger emotional regulation pathway observed in the present findings. Conversely, highly performance-oriented or excessively evaluative teaching approaches may intensify anxiety, peer comparison, or social pressure, potentially weakening the emotional and relational benefits associated with SEL experiences in PE (De Neve et al., 2023; Xing and Ge, 2025; Zeng et al., 2025). In addition, PE teachers may indirectly influence peer relationship quality by structuring collaborative activities, promoting respectful interaction, and managing group dynamics during physical activities. Accordingly, the present findings highlight that PE teachers should not be viewed merely as facilitators of physical skill development, but also as important social and emotional agents within school-based well-being processes.

The absence of a moderating effect of school climate on the peer relationship pathway suggests that improvements in peer dynamics may require more targeted or sustained social interventions beyond general climate support. While a positive school climate may create favorable conditions for social interaction, peer relationship quality in physical education settings may be influenced by additional factors such as group composition, competitive structures, and instructional practices that were not directly addressed in the present model. This finding highlights the complexity of social processes in PE contexts and suggests that peer-related outcomes may not automatically translate into enhanced mental well-being without concurrent development of emotional regulation skills. Accordingly, future interventions may need to combine emotional skill training with structured social interaction strategies to more effectively influence peer-based pathways.

At the same time, the present findings should be interpreted in light of the absence of PE-specific contextual variables within the analytical model. Physical education environments are shaped by instructional practices, teacher behaviors, activity formats, competitive structures, and classroom interaction patterns, all of which may influence how students experience emotional regulation and peer relationships during PE activities. For example, PE teachers who emphasize cooperation, emotional support, and inclusive participation may strengthen students’ emotional regulation capacities and peer bonding differently than highly performance-oriented instructional approaches. Similarly, variations in team-based versus individual activities, class organization, or teacher feedback practices may affect whether relational dynamics become psychologically supportive or socially evaluative.

Taken together, these findings indicate that SEL in physical education contributes to student mental well-being primarily by strengthening students’ capacity for emotional regulation, particularly when implemented within supportive school climates. By clarifying the relative importance of emotional and social pathways, the present study provides a more nuanced understanding of how SEL operates in PE settings and prepares the groundwork for the theoretical and practical implications discussed in the following sections.

### 6.4 Theoretical implications

This study makes several significant theoretical contributions to the evolving literature on socio-emotional learning, emotional regulation, student well-being, and school climate. Grounded in the Collaborative for Academic, Social, and Emotional Learning (CASEL) framework (Collaborative for Academic, Social, and Emotional Learning, 2020), the study validates the core proposition that SEL fosters student development across intrapersonal and interpersonal domains. However, rather than assuming equivalent effects across competencies, the present findings refine SEL theory by demonstrating that these domains may operate with different levels of functional importance within physical education (PE) contexts. In particular, the results suggest that self-management competencies—operationalized through emotional regulation—constitute a more central pathway to mental well-being than relational competencies in this domain. This differentiation advances SEL theory by emphasizing conditional and context-sensitive effects of specific competencies, rather than uniform impacts across all SEL dimensions.

The study also contributes to Self-Determination Theory (SDT) by clarifying how SEL-related competencies selectively activate psychological need fulfillment. Rather than treating SEL as a generalized motivational resource, the findings indicate that emotional regulation is particularly relevant for supporting adaptive functioning associated with competence and autonomy (Sorrenti et al., 2025). This suggests that emotional self-regulation may represent a primary mechanism through which SEL contributes to well-being in emotionally demanding learning environments such as PE. In contrast, the non-significant role of peer relationship quality—closely aligned with the SDT need for relatedness—highlights that relational processes may not uniformly translate into well-being outcomes across instructional contexts. This nuance extends SDT-informed educational models by emphasizing that the motivational relevance of specific needs may vary depending on contextual demands and developmental stages.

Another important theoretical contribution lies in the integration of Ecological Systems Theory (Bronfenbrenner, 1979) through the explicit modeling of school climate as a multilevel moderator. By demonstrating that the effectiveness of emotional regulation as a pathway to well-being depends on school-level conditions, the study reinforces Bronfenbrenner’s proposition that proximal psychological processes are embedded within institutional environments. Rather than positioning school climate as a background condition, the present findings conceptualize it as a boundary condition that regulates the strength of individual-level SEL mechanisms. This interactional perspective advances existing SEL research by bridging individual competencies with contextual affordances in a multilevel framework.

Finally, by situating the investigation within PE settings, the study extends SEL theory beyond traditional academic classrooms into learning environments characterized by heightened emotional arousal, physical engagement, and social visibility. Although prior research has examined SEL primarily within classroom instruction, comparatively limited attention has been given to its operation in PE contexts (Ciotto and Gagnon, 2018; Dyson et al., 2021; Goh and Connolly, 2020). The present findings support the generalizability of core SEL mechanisms across instructional domains while simultaneously highlighting the need for domain-sensitive theorization. This contribution aligns with emerging perspectives that conceptualize SEL as a whole-school developmental framework rather than a subject-specific intervention (Olive et al., 2021; Wilson, 2022).

In summary, this study advances SEL theory by clarifying which competencies matter most, under which contextual conditions, and within which instructional domains, reinforcing the value of multi-theoretical and multilevel approaches while avoiding assumptions of uniform competency effects.

### 6.5 Practical implications

The findings of this study offer several actionable insights for educators, school leaders, and policymakers seeking to enhance student well-being through socio-emotional learning practices. First and foremost, the results affirm the importance of integrating SEL into the PE curriculum. Often viewed primarily through the lens of physical skill development, PE also offers a dynamic and interactive context in which students can develop critical emotional competencies such as self-regulation, impulse control, and stress management. From a practical standpoint, PE classes can be deliberately structured to include brief reflective or emotion-focused activities that help students connect physical experiences with emotional awareness. Educators should intentionally embed structured SEL activities into PE sessions to help students translate physical experiences into emotional learning opportunities.

The strong and consistent mediating role of emotional regulation suggests that interventions targeting students’ ability to understand, manage, and express their emotions are particularly effective in promoting well-being. Therefore, schools should consider adopting evidence-based SEL programs that emphasize emotional awareness and self-management, such as mindfulness routines, emotion labeling exercises, or reflection circles, especially in activity-based settings like PE. Professional development initiatives should equip PE teachers with practical strategies to model and reinforce emotional regulation during instruction, as their instructional practices play a key role in shaping students’ emotional learning experiences.

Conversely, the non-significant role of peer relationship quality as a mediator suggests that while positive peer interactions are important, they may not independently enhance well-being unless coupled with strong intrapersonal emotional skills. This indicates that efforts to build social connection in schools should be complemented by emotional skills training, rather than viewed as a substitute for it. SEL initiatives are likely to yield stronger outcomes when embedded within broader school culture and climate improvement strategies, rather than being implemented as isolated curricular additions.

A key practical insight is the critical moderating role of school climate. The positive interaction between SEL and a supportive school environment suggests that SEL-related associations may be stronger when implemented in schools that foster psychological safety, trust, and inclusiveness. School leaders should therefore prioritize cultivating a positive school climate by building strong teacher-student relationships, enforcing fair discipline practices, and encouraging student voice. SEL initiatives should be embedded within broader school culture transformation strategies rather than treated as isolated interventions.

Finally, these findings call for multi-level collaboration among school administrators, PE instructors, mental health professionals, and policy designers to ensure that SEL is not only integrated into the curriculum but also supported structurally at the institutional level. By creating emotionally supportive and socially safe school environments, educators can maximize the developmental benefits of SEL and foster lasting improvements in student well-being.

### 6.6 Limitations and future studies

While this study offers valuable insights into the mechanisms linking SEL to student well-being within PE settings, several limitations must be acknowledged. First, the cross-sectional design limits causal interpretations; future research should employ longitudinal or experimental designs to verify temporal sequencing and causality. Second, reliance on self-reported data raises concerns about social desirability and common method variance, despite steps taken to ensure anonymity and scale reliability. Because all key constructs were assessed using student self-reports, future studies may benefit from incorporating multi-source data to reduce single-source bias. Incorporating multi-informant data—such as teacher ratings or peer assessments—could enhance construct validity.

Third, while the multilevel analysis accounted for school climate and several demographic control variables, the study did not incorporate additional PE-specific contextual controls such as physical activity duration, activity type, instructional practices, teacher behaviors, or class structure. These factors may vary across schools and regions and could influence students’ emotional and relational experiences during PE participation. Future studies are encouraged to integrate these PE-specific contextual variables to provide a more rigorous and contextually sensitive understanding of SEL processes within physical education environments.

## 7 Conclusion

This study advances the literature on socio-emotional learning by demonstrating how emotional regulation and peer relationship quality mediate the relationship between SEL and student well-being, and how these pathways are moderated by school climate. Grounded in the CASEL framework, Self-Determination Theory, and Ecological Systems Theory, the findings underscore that SEL is most effective when embedded in emotionally supportive school environments and when it targets both intrapersonal and interpersonal competencies. Importantly, the study extends SEL research into physical education contexts—an area often neglected—highlighting the relevance of emotional and social learning in activity-based environments. By integrating individual and contextual mechanisms into one model, this research contributes to a more nuanced understanding of how, when, and for whom SEL works. The findings offer practical implications for educators, school leaders, and policymakers aiming to cultivate emotionally intelligent, socially connected, and psychologically resilient students.

## Data Availability

The original contributions presented in the study are included in the article/supplementary material, further inquiries can be directed to the corresponding author.

## References

[ref1] Allbright T. Hough H. (2020). Measures of SEL and school climate in California. State Education Standard 20:28.

[ref2] Ayllón-Salas P. Fernández-Martín F. D. (2024). The role of social and emotional skills on adolescents’ life satisfaction and academic performance. Psychol. Soc. Educ. 16, 49–56. doi: 10.21071/pse.v16i1.16625

[ref3] Berkowitz R. Moore H. Astor R. A. Benbenishty R. (2017). A research synthesis of the associations between socioeconomic background, inequality, school climate, and academic achievement. Rev. Educ. Res. 87, 425–469. doi: 10.3102/0034654316669821

[ref4] Bronfenbrenner U. (1979). The Ecology of Human Development: Experiments by Nature and Design. Cambridge: Harvard University Press.

[ref5] Bukowski W. M. Hoza B. Boivin M. (1994). Measuring friendship quality during pre- and early adolescence: the development and psychometric properties of the friendship qualities scale. J. Soc. Pers. Relat. 11, 471–484. doi: 10.1177/0265407594113011

[ref6] Chang Y. C. Tsai Y. T. (2022). The effect of university students’ emotional intelligence, learning motivation and self-efficacy on their academic achievement—online English courses. Front. Psychol. 13:818929. doi: 10.3389/fpsyg.2022.818929, 35250754 PMC8888520

[ref7] Ciotto C. M. Gagnon A. G. (2018). Promoting social and emotional learning in physical education. J. Phys. Educ. Recreat. Dance 89, 27–33. doi: 10.1080/07303084.2018.1430625

[ref8] Collaborative for Academic, Social, and Emotional Learning. (2020). What is SEL? Available online at: https://casel.org/what-is-sel/ (Accessed June 11, 2026).

[ref9] Collie R. J. Shapka J. D. Perry N. E. (2011). Predicting teacher commitment: the impact of school climate and social–emotional learning. Psychol. Sch. 48, 1034–1048. doi: 10.1002/pits.20611

[ref10] De Neve D. Bronstein M. V. Leroy A. Truyts A. Everaert J. (2023). Emotion regulation in the classroom: a network approach to model relations among emotion regulation difficulties, engagement to learn, and relationships with peers and teachers. J. Youth Adolesc. 52, 273–286. doi: 10.1007/s10964-022-01678-2, 36180661 PMC9524346

[ref11] Demirci Z. A. Bıçakcı M. Y. Uysal B. (2022). Investigation of the effect of social emotional learning on peer relationships of adolescents. J. Educ. Fut 21, 1–13. doi: 10.30786/jef.789061

[ref12] Durlak J. A. Weissberg R. P. Dymnicki A. B. Taylor R. D. Schellinger K. B. (2011). The impact of enhancing students’ social and emotional learning: a meta-analysis of school-based universal interventions. Child Dev. 82, 405–432. doi: 10.1111/j.1467-8624.2010.01564.x, 21291449

[ref13] Dyson B. Howley D. Wright P. M. (2021). A scoping review critically examining research connecting social and emotional learning with three model-based practices in physical education: have we been doing this all along? Eur. Phys. Educ. Rev. 27, 76–95. doi: 10.1177/1356336X20923710

[ref14] Foster J. L. Louis L. Winston E. (2022). Creating conditions for social-emotional learning: an ecological framework. Theory Pract. 61, 224–235. doi: 10.1080/00405841.2022.2036059

[ref15] García-Martínez I. Gavín-Chocano Ó. Molero D. León S. P. (2023). Analysing university students’ life satisfaction through their socioemotional factors. Revista de Investigación Educativa 41, 107–124. doi: 10.6018/rie.496341

[ref16] Goh T. L. Connolly M. (2020). Efficacy of school-based SEL programs: aligning with health and physical education standards. J. Phys. Educ. Recreat. Dance 91, 16–19. doi: 10.1080/07303084.2020.1739430

[ref17] Gualda R. C. Moraleda A. Brackett M. A. (2023). Preventative initiatives to promote psychological adjustment among primary students: findings of the RULER approach in Spanish public schools. Int. J. Educ. Psychol. 12, 206–232. doi: 10.17583/ijep.10970

[ref18] Gullone E. Taffe J. (2012). The emotion regulation questionnaire for children and adolescents (ERQ–CA): a psychometric evaluation. Psychol. Assess. 24, 409–417. doi: 10.1037/a0025777, 22023559

[ref19] Hayashi A. Liew J. Aguilar S. D. Nyanamba J. M. Zhao Y. (2022). Embodied and social-emotional learning (SEL) in early childhood: situating culturally relevant SEL in Asian, African, and north American contexts. Early Educ. Dev. 33, 746–763. doi: 10.1080/10409289.2021.2024062

[ref20] Hoskins J. E. S. Schweig J. D. (2024). SEL in context: school mobility and social-emotional learning trajectories in a low-income, urban school district. Educ. Urban Soc. 56, 164–200. doi: 10.1177/00131245221106735

[ref21] Hu L. T. Bentler P. M. (1999). Cutoff criteria for fit indexes in covariance structure analysis: conventional criteria versus new alternatives. Struct. Equ. Model. 6, 1–55. doi: 10.1080/10705519909540118

[ref22] Jones T. M. Fleming C. Williford A. (2020). Racial equity in academic success: the role of school climate and social emotional learning. Child Youth Serv. Rev. 119:105623. doi: 10.1016/j.childyouth.2020.105623, 33311826 PMC7731917

[ref23] LaBelle B. (2023). Positive outcomes of a social-emotional learning program to promote student resiliency and address mental health. Contemp. Sch. Psychol. 27, 1–7. doi: 10.1007/s40688-019-00263-y

[ref24] LeBreton J. M. Moeller A. N. Wittmer J. L. S. (2023). Data aggregation in multilevel research: best practice recommendations and tools for moving forward. J. Bus. Psychol. 38, 239–258. doi: 10.1007/s10869-022-09853-9

[ref25] León B. Fernandez-Rio J. Rivera-Pérez S. Iglesias D. (2023). Cooperative learning, emotions, and academic performance in physical education: a serial multiple mediation model. Psicología Educativa. Revista de los Psicólogos de la Educación 29, 75–82. doi: 10.5093/psed2023a2

[ref26] Li X. Li Y. Hu W. Li K. Gao L. (2023). More socio-emotional regulation, more effective? Exploring social regulation of learning in collaborative argumentation among high- and low-performing groups. Metacogn. Learn. 18, 261–293. doi: 10.1007/s11409-022-09329-4

[ref27] Liang X. Li M. Wu Y. Wu X. Hou X. Sit C. H. P. (2022). A socio-ecological approach to inclusive physical education in China: a systematic review. Front. Public Health 10:902791. doi: 10.3389/fpubh.2022.902791, 35991013 PMC9382582

[ref28] Little R. J. A. Rubin D. B. (2020). Statistical Analysis with Missing data. 3rd Edn New York: Wiley.

[ref29] Low S. Van Ryzin M. J. (2024). Student-centered instruction can build social–emotional skills and peer relations: findings from a cluster-randomized trial of technology-supported cooperative learning. Sch. Psychol. 39, 672–681. doi: 10.1037/spq0000589 37902702

[ref30] McCormick M. P. Cappella E. O’Connor E. E. McClowry S. G. (2015). Context matters for social-emotional learning: examining variation in program impact by dimensions of school climate. Am. J. Community Psychol. 56, 101–119. doi: 10.1007/s10464-015-9733-z, 26099299

[ref31] Mengíbar D. L. Ripollés P. M. Lis R. Delafuente A. G. Beitia M. D. S. Seisdedos R. T. . (2025). Socioemotional learning in physical education: a systematic review in secondary school. Retos 63, 74–90. doi: 10.35542/osf.io/ap2jr

[ref32] Mondi C. F. Giovanelli A. Reynolds A. J. (2021). Fostering socio-emotional learning through early childhood intervention. Int. J. Child Care Educ. Policy 15, 1–43. doi: 10.1186/s40723-021-00084-8, 39935621 PMC11812064

[ref33] Morcillo-Martínez A. Gómez-Barreto I. M. Infantes-Paniagua Á. Fernández-Bustos J. G. (2026). Socio-emotional skills and physical activity in primary and secondary education students. J. Sch. Health 96:e70152. doi: 10.1111/josh.70152, 41988739 PMC13084471

[ref34] Moreira A. L. Yunes M. Â. M. Nascimento C. R. R. Bedin L. M. (2021). Children’s subjective well-being, peer relationships and resilience: an integrative literature review. Child Indic. Res. 14, 1723–1742. doi: 10.1007/s12187-021-09843-y

[ref35] Niemiec C. P. Ryan R. M. (2009). Autonomy, competence, and relatedness in the classroom: applying self-determination theory to educational practice. Theory Res. Educ. 7, 133–144. doi: 10.1177/1477878509104318

[ref36] Olive C. Gaudreault K. L. Lucero A. (2021). Strategies for implementing social-emotional learning in adapted physical education. Teach. Except. Child. 54, 63–69. doi: 10.1177/00400599211046279

[ref37] Pérez B. R. Bahamon Muneton M. J. (2023). The socio-emotional dimension in education: a systematic review. Issues Educ. Res. 33, 307–326.

[ref38] Rivers S. E. Brackett M. A. Reyes M. R. Elbertson N. A. Salovey P. (2013). Improving the social and emotional climate of classrooms: a clustered randomized controlled trial testing the RULER approach. Prev. Sci. 14, 77–87. doi: 10.1007/s11121-012-0305-2, 23188089

[ref39] Rodríguez S. González-Suárez R. Vieites T. Piñeiro I. Díaz-Freire F. M. (2022). Self-regulation and students’ well-being: a systematic review (2010–2020). Sustainability 14:2346. doi: 10.3390/su14042346

[ref40] Ryan R. M. Deci E. L. (2000). Self-determination theory and the facilitation of intrinsic motivation, social development, and well-being. Am. Psychol. 55, 68–78. doi: 10.1037/0003-066X.55.1.68, 11392867

[ref41] Serey A. K. Martínez-Líbano J. Barahona-Fuentes G. (2025). Emotional regulation and subjective well-being in adolescents: a systematic review. Ment. Health: Glob. Chall. 8, 14–26. doi: 10.56508/mhgcj.v8i1.240, 42109276

[ref42] Simion A. (2023). The impact of socio-emotional learning (SEL) on academic evaluation in higher education. Education 21, 109–117. doi: 10.24193/ed21.2023.24.11

[ref43] Sindiani M. Schroeder H. B. Dunsky A. (2025). Social-emotional learning in physical education classes at elementary schools. Front. Psychol. 16:1499240. doi: 10.3389/fpsyg.2025.1499240, 40256436 PMC12007113

[ref44] Slee P. T. Skrzypiec G. (2016). Well-Being, Positive Peer Relations and Bullying in School Settings. Cham: Springer.

[ref45] Sorrenti G. Zölitz U. Ribeaud D. Eisner M. (2025). The causal impact of socio-emotional skills training on educational success. Rev. Econ. Stud. 92, 506–552. doi: 10.1093/restud/rdae018

[ref46] Taylor R. D. Oberle E. Durlak J. A. Weissberg R. P. (2017). Promoting positive youth development through school-based social and emotional learning interventions: a meta-analysis of follow-up effects. Child Dev. 88, 1156–1171. doi: 10.1111/cdev.12864, 28685826

[ref47] Tennant R. Hiller L. Fishwick R. Platt S. Joseph S. Weich S. . (2007). The Warwick-Edinburgh mental well-being scale (WEMWBS): development and UK validation. Health Qual. Life Outcomes 5:63. doi: 10.1186/1477-7525-5-63, 18042300 PMC2222612

[ref48] Törmänen T. Järvenoja H. Saqr M. Malmberg J. Järvelä S. (2023). Affective states and regulation of learning during socio-emotional interactions in secondary school collaborative groups. Br. J. Educ. Psychol. 93, 48–70. doi: 10.1111/bjep.12525, 35748024

[ref49] Trach J. Lee M. Hymel S. (2018). A social-ecological approach to addressing emotional and behavioral problems in schools: focusing on group processes and social dynamics. J. Emot. Behav. Disord. 26, 11–20. doi: 10.1177/1063426617742346

[ref50] Váradi J. (2022). A review of the literature on the relationship of music education to the development of socio-emotional learning. SAGE Open 12:21582440211068501. doi: 10.1177/21582440211068501

[ref51] Wang M. T. Degol J. L. (2016). School climate: a review of the construct, measurement, and impact on student outcomes. Educ. Psychol. Rev. 28, 315–352. doi: 10.1007/s10648-015-9319-1

[ref52] Wang M. Zeng Q. Wu W. Guo K. Xie R. (2026). How physical exercise influences children’s social-emotional competence: an empirical study based on a chain mediation model. BMC Psychol 14:788. doi: 10.1186/s40359-026-04540-3 41987331 PMC13217644

[ref53] Wilson Z. (2022). Physical Activity and How it Influences Mental Health and SEL. Allentown: Cedar Crest College.

[ref54] Winardi M. A. Prentice C. Weaven S. (2022). Systematic literature review on emotional intelligence and conflict management. J. Glob. Scholars Market. Sci. 32, 372–397. doi: 10.1080/21639159.2020.1808847

[ref55] Xing Z. Ge C. (2025). The relationship between physical exercise and social adjustment in Chinese university students: the sequential mediating effect of peer attachment and self-esteem. Front. Psychol. 16:1525811. doi: 10.3389/fpsyg.2025.1525811, 40432802 PMC12106527

[ref56] Yang C. Bear G. G. May H. (2018). Multilevel associations between school-wide social–emotional learning approach and student engagement across elementary, middle, and high schools. Sch. Psychol. Rev. 47, 45–61. doi: 10.17105/SPR-2017-0003.V47-1

[ref57] Yang C. Chan M. K. Ma T. L. (2020). School-wide social emotional learning (SEL) and bullying victimization: moderating role of school climate in elementary, middle, and high schools. J. Sch. Psychol. 82, 49–69. doi: 10.1016/j.jsp.2020.08.002, 32988463

[ref58] Yang C. Chen C. Lin X. Chan M. K. (2021). School-wide social emotional learning and cyberbullying victimization among middle and high school students: moderating role of school climate. School Psychol. 36, 75–85. doi: 10.1037/spq0000423

[ref59] You Y. Zhang S. Zhang W. Mao Y. (2023). The impact of social and emotional learning on students' bullying behavior: serial mediation of social and emotional competence and peer relationship. Psychol. Sch. 60, 3694–3706. doi: 10.1002/pits.22947

[ref60] Zeng Q. Wen P. Guo K. (2025). The impact of physical exercise on adolescents’ social-emotional competence: the chain mediating role of social support and peer relationships. PLoS One 20:e0334587. doi: 10.1371/journal.pone.0334587, 41259296 PMC12629442

[ref61] Zheng J. (2022). The temporal Changes of Emotions and their Relationships to Self-Regulated Learning: A multi-Study Examination (Doctoral dissertation). Montreal: McGill University.

